# A 3-year-old boy with a depressed, whitish lesion on the left buttock

**DOI:** 10.4103/0256-4947.51799

**Published:** 2009

**Authors:** Mohammed Al Jasser, Sultan Al-Khenaizan

**Affiliations:** Division of Dermatology, Department of Medicine, King Fahad National Guard Hospital, King Abdulaziz Medical City

A 3-year-old Saudi boy with severe atopic dermatitis presented with a depressed white area on the left buttock. Three weeks previously, the parents noticed this insidious onset, asymptomatic, whitish depression in the left gluteal area. His medical history was not significant except for a chronic history of severe atopic dermatitis. On physical examination, there was a well-defined, round hypopigmented, atrophic plaque on the left buttock (Figure 1). There were no other areas of atrophy with normal fat distribution elsewhere on the body.

**Figure 1:**
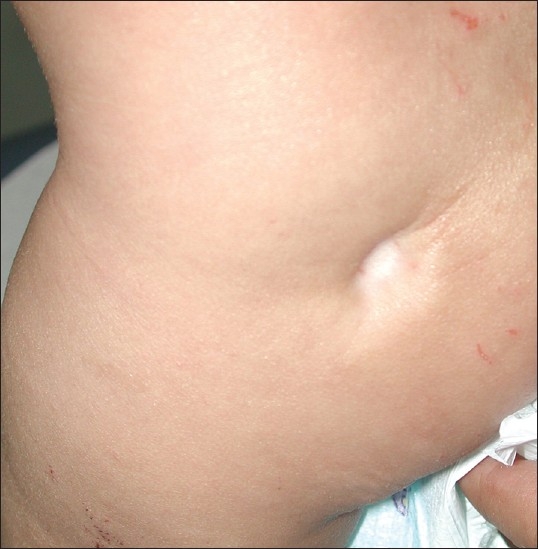
A depressed, whitish lesion on the buttocks.

What is your diagnosis?

FOR THE ANSWER, VISIT:

http://www.saudiannals.net

## Diagnosis: Localized lipoatrophy due to intramuscular steroid injection

**Figure 2:**
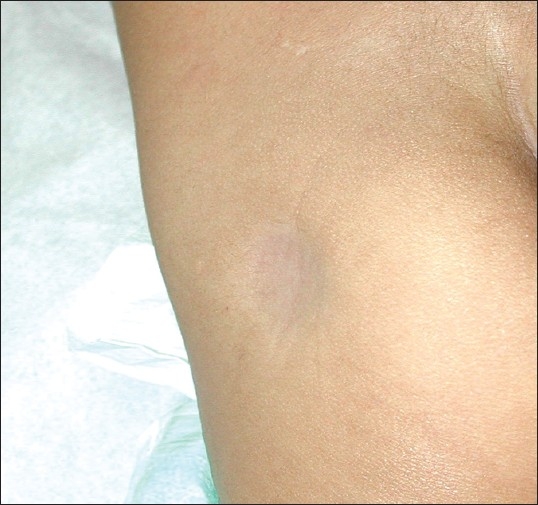
Improvement at 6-months follow-up.

On inquiry, this was the site of an intramuscular injection of triamcinolone acetonide (Kenacort, Bristo-Myers-Squibb) given 6 weeks ago to control a flare of his eczema. A clinical diagnosis of steroid-induced lipoatrophy and hypopigmentation was made. Reassurance was given and simple observation was advised. In a follow-up visit 6 months later, the atrophy had significantly improved but did not completely resolve (Figure 2).

## DISCUSSION

Lipoatrophy (LA) can be congenital or aquired.^1^ Aquired LA is classified into idiopathic (primary) and secondary types.^1^ One cause of secondary LA is iatrogenic injury from subcutaneous, intramuscular, or intradermal injections.^1^ Injected substances include human growth hormone, steroids, insulin, and antibiotics.^2^–^6^

There are few reports of LA secondary to intramuscular steroid injection.^1^,^2^,^7^,^8^ Except for one boy all were females.^1^ This predominance might be because more adipose tissues are available for damage in females. Dahl et al found that 8 out of 16 patients had localized LA due to steroid injections.^7^ Cutaneous lesions were well-demarcated, oval, flesh-colored or faint erythematous depressions of variable sizes.^7^ Buttocks and arms were the most commonly affected, probably because these are the most commonly injected sites.^7^ Associated medical conditions were not prominent in any patient.^7^ Aviles-Izquierdo et al reported a patient who developed LA in both buttocks after injection in the right buttock.^1^ Laboratory investigations are usually normal in patients with localized LA.^7^ Histopathologic features include small fat lobules, with a reduced number of small to medium-sized lipocytes within fat lobules.^7^,^9^ Prominent infiltration of large granular or vacuolated macrophages can also be seen.^9^ Zalla et al hypothesized that steroid injections stimulate a macrophage response, with subsequent tumor necrosis factor alpha-induced regression of lipocytes in the neighboring fat lobules.^9^ However, it is unclear whether the macrophages are the cause or the result of the LA process.^7^

To minimize steroid-induced LA incidence, physicians should resort to the oral route whenever possible.

Intramuscular injections should be only given when justified by intolerance or compliance problems. Many basic rules were suggested by Friedman to prevent steroid-induced LA.^10^ The gluteal area should be of sufficient size.^10^ If not, the injection should be given elsewhere. The patient's gluteal muscles should be relaxed so that the needle does not end up in the subcutaneous tissue.^10^ An experienced, qualified individual (nurse or physician) should give the injection deeply into the muscle.^10^ Steroid-induced LA usually spontaneously resolves or improves in 2-4 months.^1^ If lesions persist and are of cosmetic concern many surgical procedures can be suggested to improve the appearance. These include antologous fat transplantation and the use of different fillers to fill the defect.^11^,^12^
